# Influence of Reclaimed Water on the Visual Quality of Automotive Coating

**DOI:** 10.3390/ma17215382

**Published:** 2024-11-04

**Authors:** Piotr Woźniak, Marek Gryta

**Affiliations:** Faculty of Chemical Technology and Engineering, West Pomeranian University of Technology in Szczecin, ul. Pułaskiego 10, 70-322 Szczecin, Poland; piotr.wozniak@zut.edu.pl

**Keywords:** automotive coatings, car wash, water reuse, water reclamation, ultrafiltration

## Abstract

In the present study, the possibility of recovering water in a car wash station was presented. The resistance of automotive coatings to washing water recovered at 50% and 70% from wastewater generated at car wash was tested. Wastewater treatment was carried out by ultrafiltration (UF) using tubular polyvinylidene fluoride (PVDF) membranes (100 and 200 kDa) manufactured by the PCI company. The membranes retained oil contamination, suspended solids, and over 60% of surfactants. For comparison, the 0.5% Turbo Active Green solution, used at professional car washes, was also applied in paint resistance studies. The tested solutions washed the painted surfaces of samples taken from car doors for 8 days. The resistance of automotive coatings to washing solutions was assessed by measuring gloss, Log Haze, RIQ, and Rspec parameters. Scratch resistance was also assessed. The results obtained in the current study indicated that the use of water recovered from wastewater did not deteriorate the quality of the car paint coating.

## 1. Introduction

Cars need to be washed periodically, which results in an annual worldwide water consumption of millions of cubic meters [1,2,3]. For this reason, regulations are increasingly being introduced to limit water consumption in washing facilities, particularly in water-scarce areas [4,5]. Obviously, the amount of washing water consumed by car washes depends on the technology used. Commercial car wash operations are realised by applying different automatic or manual systems, using approximately 150–350 L of water per car [6,7]. In Europe, some countries limit water consumption to 60–70 L per car and require to achieve a high water recovery rate [8,9].

Commercial carwash stations are typically equipped with a settling tank and an oil separator. The quality of the water treated in this way is too low to be recycled for car washing. Therefore, several additional systems are designed and tested for the treatment and reuse of the wastewater generated in carwash stations. For these purposes, multi-stage filtration through sand and activated carbon beds [5,10], membrane separation [2,11], chemical methods like coagulation and flocculation [12,13], electrocoagulation [14,15], and biological treatment [16,17] are used. Many of these methods produce purified water with parameters even better than tap water, which is usually fed to car washes.

Car washing is carried out in several stages, and at each stage, water from different purities can be used. Demineralised water (reverse osmosis separation) is used in the final rinsing stage (spot-free water) to achieve a shiny car finish without streaks or traces of salt deposits. At the initial process stage, for pre-wash and foam washing, there is no need for such pure water; hence, reclaimed water can be used for this [2,3]. Furthermore, multi-stage cleaning systems are too complex and expensive to be applied in small hand touchless car washes. Therefore, this study investigated the possibility of recycling water treated in a simple ultrafiltration (UF) system.

Small touchless car washes are popular in many countries, and there are several in every Polish city. These are self-service car washes, where the customer can choose and apply a washing programme, usually including water rinsing, foam washing, and final water rinsing. A final option, including rinsing with osmotic water and waxing for paint protection, is also available. Water demineralised by reverse osmosis is used for these final stages, but reclaimed water can be used for the initial two stages. Washing water and cleaning agents are sprayed onto the car using high-pressure nozzles (5–10 MPa), and therefore, reclaimed water must be free of suspended solids and microorganisms [2,18]. This degree of water purification can be achieved using the UF process [19,20].

The UF process makes it possible to separate the contaminants present in the effluent removed from car washing. The permeate obtained contains mainly water and part of the cleaning agents used at the car wash, which are retained in 60–80% [20]. The part of the cleaning agents remaining in the permeate can facilitate the removal of contaminants from the car surface. The reclaimed water may also contain traces of substances formed in the settling tank, e.g., aggressive products of the microorganisms’ metabolisms [21], whose effects on paints are not known. Moreover, hydrolytic degradation of car coatings was found to be caused by enzymes existing in bird droppings [22]. This indicates that many components present in the environment can have an impact on the appearance of a car’s body during its service life. To investigate whether such an impact is also caused by reclaimed water, the surfaces of car body components were washed for several days. Some works have presented the long-term performance of water purification systems in car wash stations [8,23]. However, the effect of reclaimed water on the durability of automotive paints has not yet been studied.

In modern cars, paint resistance is provided by the external clearcoat layer (40–50 μm) covering the coloured basecoat (15–20 μm), which is deposited on the corrosion protection layer (primer and electrocoat, 40–60 μm) [22,24]. The automotive paint forming the basecoat has little abrasion resistance, hence the scratch-free finishes provided by clearcoat. Scratch events occur from very small (micron size) to very large, and they appear white due to fractures in the clearcoat [24]. Pores and oxidation products can form on the paint surface due to negative environmental influences, resulting in reduced gloss of the paint and reduced scratch resistance [25,26].

Manufacturers design the products to have highly reflective car body panels. Gloss, distinctness of image (DOI), image fuzziness (Haze), and waviness (orange peel) measurements are used to assess surface finish quality [24,27,28]. Typical gloss meter systems are designed around standard measurement angles of 20° for high-gloss surfaces, 60° for semi-gloss surfaces, and 85° for matte surfaces [29,30]. The defined reference points of gloss unit (GU) in the standards are a 0 GU (zero reflection) and a 100 GU reference [31]. DOI quantifies the spread of light reflected at the specular angle. It gives an indication of how sharp a surface reflection is likely to be. Haze refers to a cloudy or milky appearance, also due to scattering of light. Other industry standards have been developed by Rhopoint Instruments company, two of which are peak specular reflectance (Rspec) for smoothness quantification and the reflected image quality (RIQ) parameter [29]. Rspec is very sensitive to surface texture and can be used to identify subtle differences in smooth surfaces. RIQ is used to quantify effects such as orange peel and surface waviness. This new parameter provides higher resolution results compared to DOI measurement and better mimics human perception of surface texture, especially on high-quality finishes such as automotive.

The effect of using reclaimed water on changes in the above parameters, as well as the scratch resistance of clearcoat, was investigated in the study. The tests were conducted to determine whether reclaimed water is more detrimental to the visual quality of car paint than the commercial washing solution used in the car wash.

## 2. Materials and Methods

### 2.1. Washing Water

The permeates obtained during ultrafiltration of wastewater collected from touchless car washes were used for the study. The UF process was carried out using polyvinylidene fluoride (PVDF) tubular membranes FP100 (100 kDa) and FP200 (200 kDa), manufactured by PCI (Kostrzyn, Poland). The wastewater separation process is described in work [20]. The UF process was carried out using a 50% and 75% water recovery rate, obtaining the UF permeates of the composition shown in Table 1 (Permeate 50% and Permeate 75%). In addition to the permeates, in presented studies, a 0.5% Turbo Active Green Foam solution (EuroEcol, Łódź, Poland) was also used for comparison. At the car wash stations from which the wastewater was collected, this cleaning agent was used to prepare a washing solution.

The Hach cuvette tests (Hach Lange, Wrocław, Poland) were used to determine the concentration of surfactants (LCK 334-nonionic, LCK 344-anionic) and chemical oxygen demand (COD) (LCK 1014). The biological oxygen demand (BOD) was determined using test LCK 555 and the total P and total N by using LCK 348 and LCK 238 tests, respectively.

### 2.2. Experimental Installation

**Figure 1:**
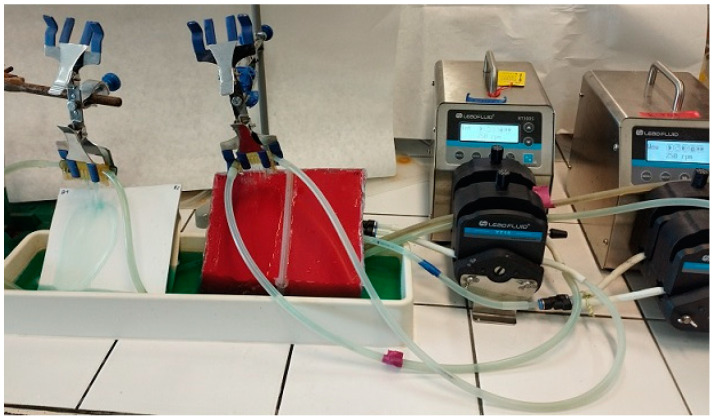
The image of the experimental installation.

The study evaluated the effect of prolonged contact with the cleaning solution on the changes in the visual quality of the automotive paint finishes. The design of the experimental setup is shown in Figure 1. The tested car body samples mounted at an angle of approximately 40° were poured with wash water for 8 days. Washing water (2 L) was pumped by a peristaltic pump (660 mL/min) to the top of the sample and flowed in a film (5–6 cm wide, 13–15 cm long) to the tank from which it was circulated.

For the study, car body samples were cut from the metal front doors of two passenger cars and painted in red (R) and cherry (CH). The CH sample had a metallic coating, which can interfere with measurements of some parameters [29]. To improve fuel economy for vehicle body construction, lighter materials such as polymers were applied in addition to steel. The polymer materials were used to make white-painted truck doors, from which samples labelled W were taken.

Several samples were used for the study, with the starting parameters (Rhopoint IQ measurements) shown in Table 2. Of the coating types tested, red paint was the least resistant to the damaging effects of the environment. For this reason, more R samples were used in the study than the others. The samples were obtained from the cars after 4–5 years of use. As cars are used over time, the amount of damage to the clearcoat layer increases, which intensifies the negative environmental impact [21]. This property also allows a better assessment of the impact of reclaimed water.

Before performing the tests, the samples taken from the cars were cleaned. They were rinsed with distilled water and finely cleaned with isopropanol. The constituents of the reclaimed water could deposit on the samples; therefore, their surfaces were also rinsed with isopropanol each day before measurements were taken. Measurements with the Rhopoint IQ were taken once a day, collecting 30 datasets from a 3 × 7 cm area. The samples were divided into fields (Figure 1), and the Turbo Active Green solution flowed 3–4 cm from the surface over which the reclaimed water flowed, allowing the effect of both washing solutions to be studied for clearcoat with similar properties.

In the case of the abrasion resistance test, the alcohol-wetted samples were additionally scratched 10 times with filter paper. Alcohol was wiped off by moving the paper by hand on both sides of the sample without applying additional pressure. It was assumed that similar friction conditions were achieved and that the degree of surface scratching was only dependent on the performance of the clearcoat, which could change with prolonged contact with wash solutions.

### 2.3. Measuring Equipment

The quality of surface appearance was analysed using a glossmeter/goniophotometer Rhopoint IQ (Rhopoint Instruments Ltd., St Leonards on Sea, UK). The instrument uses a standard optical configuration at 60° for semi-gloss surfaces and 85° for matte surfaces. Moreover, at 20°, the Rhopoint IQ uses a diode array to measure the distribution of the reflected light at +/−7.25° from the specular angle of reflection in steps of 0.02832°. The Rhopoint IQ uses a 512-element linear diode array, which profiles reflected light in a large arc from 14° to 27°. The measures at 20° allow the instrument to calculate Haze, DOI, RIQ and Rspec parameters.

Haze describes the milky halo or bloom seen on high gloss surfaces. When measuring haze values, higher numbers indicate a lower-quality surface. The IQ instrument can display the natural haze value or Log Haze value. Log Haze is commonly quoted for paints and coatings as this scale has better corroboration with human perception of surface quality [29]. The Log Haze unit is calculated from the relationship:

Log Haze = 1285 (log10((Haze/20) + 1))(1)

The Rhopoint IQ measures the DOI (or improved RIQ parameter) of a surface by quantifying the way a reflected measurement beam is spread and distorted around the specular angle. The DOI value of a surface is a number between 01 and 100; a surface that exhibits a perfect undistorted image returns a value of 100, and as the value decreases, the image becomes less discernible. However, two highly reflective surfaces that have very small changes in orange peel or texture will show very little or no change in DOI due to the way that it is calculated but will appear quite different visually. When using the RIQ option, a greater differentiation is achieved. The RIQ value of a surface is also a number between 0 and 100; a surface that exhibits a perfect undistorted image returns a value of 100; as the values decrease, higher surface texture is present, and the image sharpness is reduced [29].

Rspec is the peak reflectance measured over a very narrow angle in the specular direction (+/−) 0.0991°. Waviness or rippling on a surface acts as a concave or convex reflector deflecting light around the specular angle. When Rspec is equal to the gloss, the surface is smooth. The RSpec parameter drops as the texture becomes apparent.

The pH of each solution was measured using a 6P Ultrameter (Myron L Company, Carlsbad, CA, USA).

A Stemi 508 optical microscope (Carl Zeiss, Oberkochen, Germany) and an OLS 5100 (Olympus, Hamburg, Germany) laser scanning microscope (LSM) were used to observe the surfaces of the tested samples. The LSM microscope software was used to measure the surface roughness parameters: root mean square height (Sq) and arithmetic mean height (Sa). The surface of filter paper was observed using an SU8020 (Hitachi High Technologies Co., Tokyo, Japan) scanning electron microscope (SEM).

## 3. Results

### 3.1. Long-Term Washing

**Figure 2:**
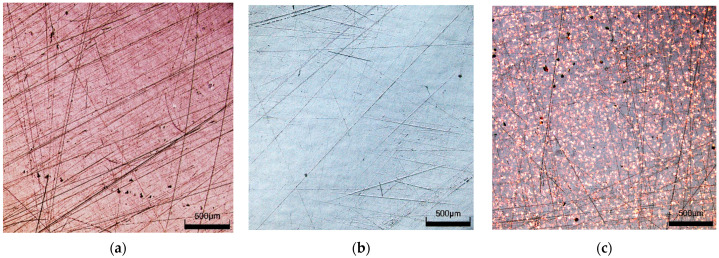
LSM images of tested non-washed samples: (**a**) R#1, (**b**) W#1, (**c**) CH#1.

In the visual assessment, the samples taken from the car doors were glossy and showed little difference in light images reflected from a surface. The samples covered with metallic paint (CH) were the most glossy and, in reflection, showed a clear image. The light image on the surface of the sample taken from the polymer door (W) was more fuzzy, and an orange peel was visible. LSM microscope observations showed that several scratches were present on the surface of the samples after 4–5 years of use of the cars, especially on the sample from the red door (Figure 2a). A similar scratch structure in the clearcoat layer was presented in work [26].

The presence of scratches affects the roughness values and paint parameters tested in the study [29]. It has been indicated that with an increase in roughness, the results of gloss values decrease rapidly [31]. The roughness values obtained for the samples tested are shown in Table 3. Higher values were obtained before the washing process, especially for the CH sample coated with metallic paint (Sq = 8.51 μm and Sa = 5.27 μm). The roughness values decreased after 8 days (190 h) of washing with the test liquids. During car operation, due to weathering degradation, i.e., hydrolytic and photodegradation, small molecules and shorter polymeric chains are produced, which increases roughness [21,32]. The washing liquids were probably able to wash away these products, and, as a result, lower roughness values for washed samples were recorded.

**Figure 3:**
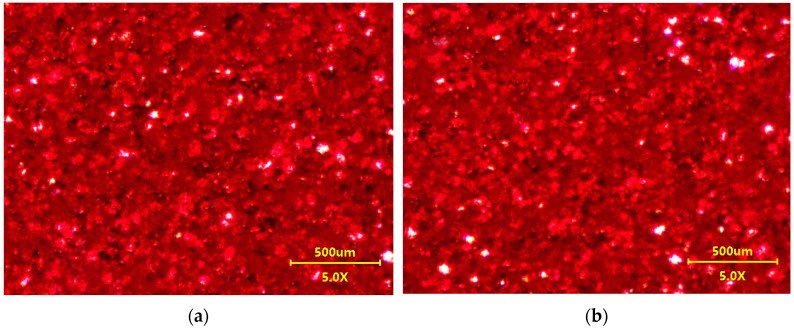
Microscopic images of surface CH#2 sample covered with metallic coating: (**a**) non-washed, (**b**) after 8 days washing with Permeate 50%.

**Figure 4:**
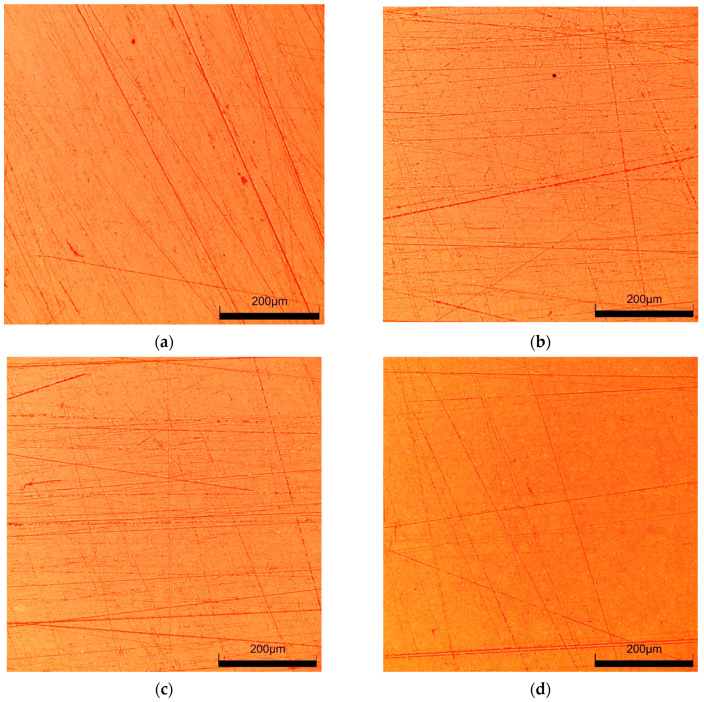
LSM images of R samples surface: (**a**) R#1-non washed, (**b**) R#1-washed by Turbo Active Green, (**c**) R#2-washed by Permeate 50%, (**d**) R#3-washed by Permeate 75%.

Despite significant changes in roughness, microscopic observations did not show significant changes in surface structure. The image of the CH samples before and after washing was similar (Figure 3). The presence of scratches on the clearcoat surface could facilitate the degradation of polymer coatings [28]. However, LSM images did not show significant changes in the scratch structure (Figure 4). This result indicates that both the Turbo Active Green solution and the water recycled from the car wash wastewater did not damage the clearcoat or increase the scratches. In contrast, after 300 h of exposure to biological substances, several local defects, as well as a decreased appearance on the clearcoat surface, were observed [22]. This result confirmed that it is important to wash the car to remove bird droppings and insects.

**Figure 5:**
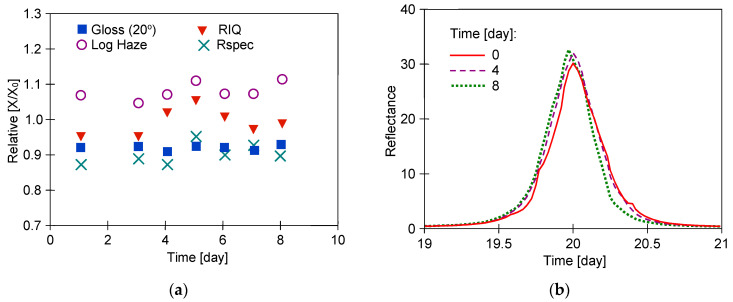

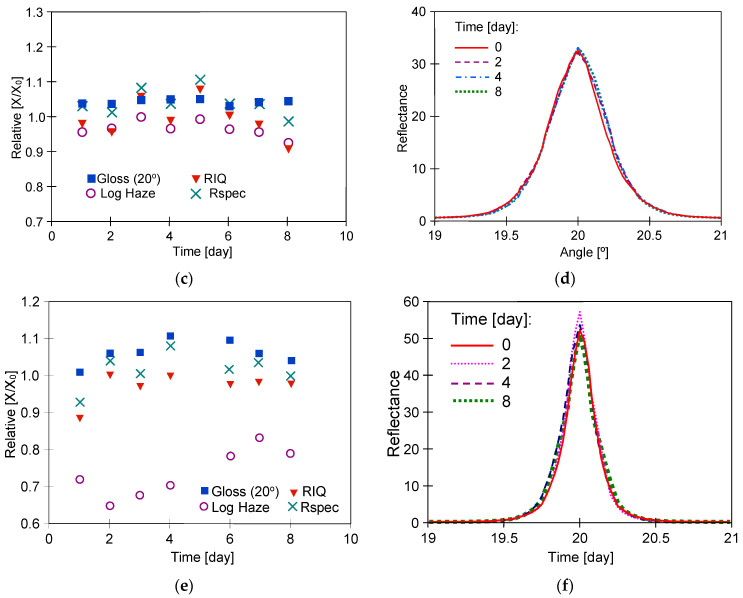
The result of car body samples washing with 0.5% Turbo Active Green solution. Samples: (**a**,**b**)—R#1, (**c**,**d**)—W#1, and (**e**,**f**)—CH#1. Goniophotometric profiles: (**b**,**d**,**f**).

**Figure 6:**
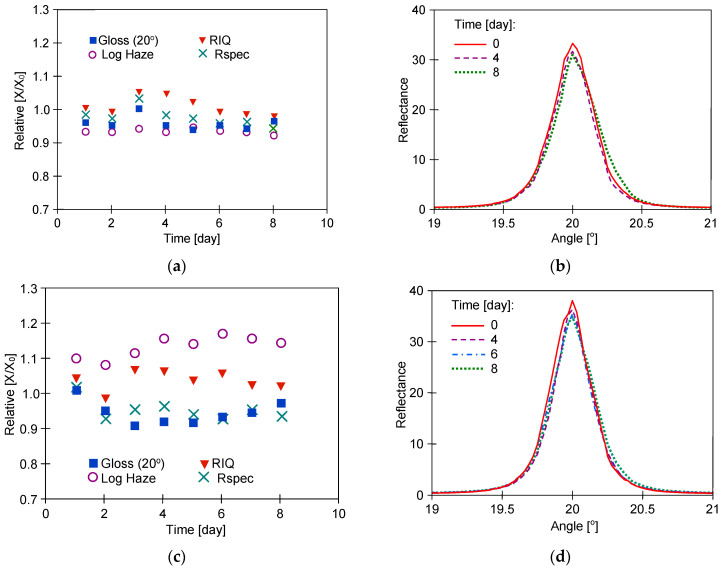
The results R samples washing with the UF permeates: (**a**,**b**) R#2-Permeate 50%, (**c**,**d**) R#3-Permeate 75%. Goniophotometric profiles: (**b**,**d**).

**Figure 7:**
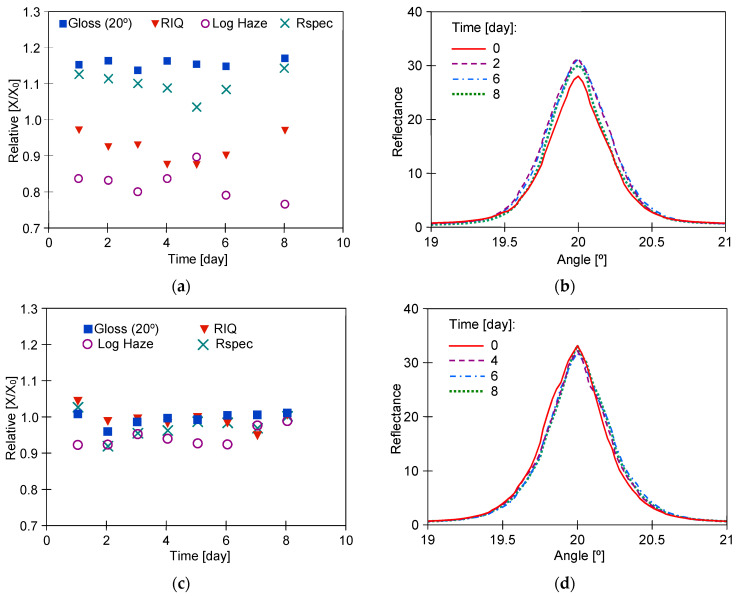
The results of W samples washing with the UF permeates: (**a**,**b**) W#2—Permeate 50%, (**c**,**d**) W#3—Permeate 75%. Goniophotometric profiles: (**b**,**d**).

The gloss values obtained for measurements at 60° were above 70 GU (Table 2), indicating that the samples tested are of the high gloss type. In this case, it is recommended that the analysis be performed at 20°, which improves accuracy and resolution on high gloss and metallic samples [29,33]. The results of the measurements for the samples taken from the different door locations differed by more than 10 per cent; hence, the relative changes in the values of the measured parameters (X/X_0_) are shown in Figure 5, Figure 6 and Figure 7.

Coatings of car bodies are frequently modified with nanoparticles, and the acrylic base coating or the metalised interlayer coating is popularly used. Unfortunately, exposing cars to environmental impacts resulted in the ageing of the coating material, which caused the corrosion of polymer coatings [28]. The acrylic coatings aged climatically for 2 years showed significant destruction of their surface in the form of microcrackings, craters, and etchings [21]. These processes influenced the surface topography, resulting in a gloss decrease from 92 to 72 GU. Similar gloss values, in the range of 70–80 GU, were obtained for samples taken from cars after more than 4 years of use (Table 2).

Obviously, car washing can cause both mechanical and chemical damage to polymer coatings [24,25,33]. This is related to the fact that cleaning agents contain many chemicals in addition to detergents [34]. The active foams commonly used at car washes are alkaline (Table 1, pH values), which helps to wash away oils and grease. However, alkalis can accelerate the degradation of automotive coatings [21]; hence, active foams deposited on the car surface should be rinsed off after a few minutes.

In the present work, the samples were in contact with the 0.5% Turbo Active Green solution for 8 days, which resulted in minor changes in the measured parameters, such as an improvement in haze (Figure 5). The measured gloss values for samples R#1 and W#1 on consecutive days were almost constant, with progressively small changes in the other parameters. In most cases of the samples tested, the larger changes occurred after the first day of testing. This was probably related to the leaching of the preservatives from the clearcoat (e.g., car wax), which was applied several times during car service life. Car waxes are used not only to protect the body of the car but also to improve the gloss and visual appearance of the car coating. Hence, their removal results in a decrease in gloss values (X/X_0_ < 1) and an increase in the Log Haze parameter (X/X_0_ > 1). In the case of Turbo Active Green solution, the image blurring increased slightly when washing sample R#1 (Figure 5a); however, gloss improved as a result of reduced roughness (Table 3). Alkaline liquids have a strong effect on polymers [34]; hence, they can remove the resulting degradation products from their surface. This was probably the reason for the slight improvement in the surface quality parameters of samples W#1 (Figure 5c) and CH#1 (Figure 5e). Nevertheless, the progressive changes in surface structure were small and similar shapes of goniophotometric profiles were obtained in each case (Figure 5).

The testing of a sample covered with a metallic coating (CH) showed the lowest values of Log Haze (Table 2), indicating the good performance of this coating. Reflection haze is caused by micro-texture on a surface, which causes a small amount of light to be reflected adjacent to the gloss angle. For metallic paint, a certain amount of diffuse light is reflected from metallic particles within the material. This diffuse light exaggerates the haze signal for these surfaces, causing higher-than-expected readings. In Rhopoint IQ, this error was eliminated by a compensation procedure [29]. The better surface quality of the CH sample is also evidenced by the higher-shaped reflectance profiles (Figure 5f). The more narrow and higher the goniophotometric profile is, the closer the gloss is to 100%. A widening of this profile means that the influence of haze increases [29].

**Figure 8:**
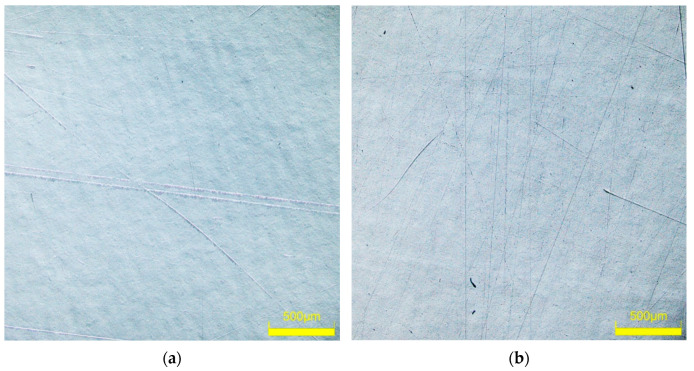
LSM images surface of W#2 sample: (**a**) non-washed, (**b**) after 8 days washing with Permeate 50%.

In the next stage of the current study, the UF permeate obtained from the wastewater (Table 1) was used to wash the samples. Wastewater was taken from car washes where alkaline Turbo Active Green Foam was used. The UF membranes do not retain low molecular weight compounds like NaOH; hence, the pH values of the tested solutions were similar (pH = 8.5–8.7). The membranes used for UF retained more than 60% of the surfactants, and their residues were present in the permeates. The P content in the collected wastewater was 12–14.5 mg/L [34]. The Turbo Active Green solution contained no phosphate; hence, the P detected in the permeates came from the removed car pollutants. To determine whether such compositional differences could have caused changes in the structure of the clearcoat layer, the 8-day washing tests were carried out. The effects of washing the samples with water recycled from wastewater (50% and 75%) were presented in Figure 6, Figure 7 and Figure 8.

For samples called ‘R’, greater changes in the parameters studied were observed for the Permeate 75% (Figure 6c), while the opposite results were obtained for W samples (Figure 7a). Moreover, Permeate 75% had a similar composition to the Permeate 50% (Table 1). These results indicate that the changes in clearcoat properties were not significantly affected by the degree of water recovery from the wastewater. In addition, the LSM images obtained for both permeate types showed no differences, and the observed surface structure of the washed ‘R’ samples was similar to that of the unwashed samples (Figure 4). It can, therefore, be assumed that the differences in the measurements obtained are due to the differences in the initial surface properties of the samples used (Table 2).

The samples called ‘W’ taken from the polymer door showed the greatest distortion of the reflected image. Under the influence of long-term washing, the surface properties improved, and the Log Haze parameter decreased, especially for sample W#2, which also increased the goniophotometric shape (Figure 7b). LSM microscope observations confirmed that, as a result of sample W washing, the orange peel decreased significantly (Figure 8), which resulted in a reduction of the Log Haze value, as also shown in another paper [29].

**Figure 9:**
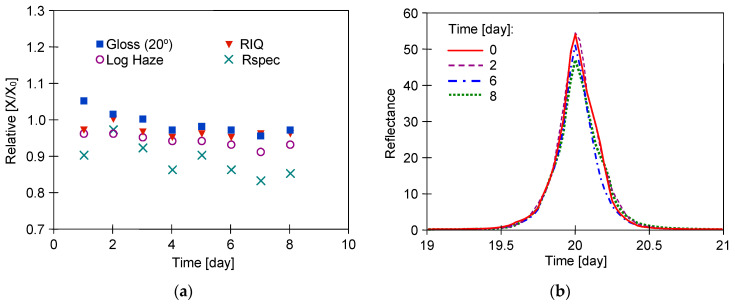
The results of CH#2 sample washing with Permeate 50%. (**a**) changes during 8 days of washing, (**b**) goniophotometric profiles.

Almost constant values of the parameters were obtained for the sample with metallic paint (CH#2), confirming the good resistance of this type of paint (Figure 9). As a result, the surface image before and after 8 days of washing was similar (Figure 3).

Due to the alkaline properties (pH > 8.5) of active foams, it is recommended that they should only be in contact with the car body for a few minutes during washing. Therefore, extending the contact time in the tests to 8 days could have caused damage to the car paint. Despite this, no significant damage to the clearcoat was found in the performed tests, and even some of the parameters tested, such as Log Haze, improved slightly. During the study, each measurement was repeated several times, moving the Rhopoint IQ apparatus by 3–5 mm across the measured area. Despite such small steps, the values obtained often differed by more than 10%, resulting in significant standard deviation values calculated for 30 repetitions (Table 2). These discrepancies were observed for all types of samples tested. Therefore, despite slight differences in individual measurements, it can be concluded that the solutions tested had a similar effect on the samples used. Therefore, it can also be concluded that the results obtained for samples washed with reclaimed water were similar to those obtained for samples washed with Turbo Active Green solution.

### 3.2. Scratch Resistance Test

**Figure 10:**
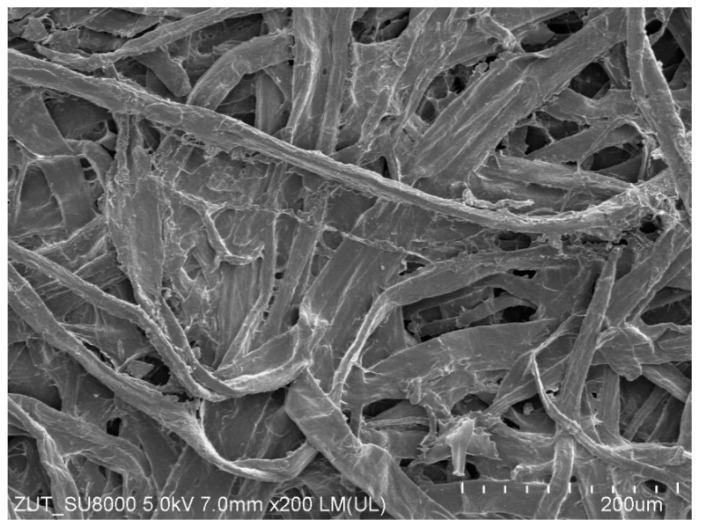
SEM image surface of filter paper applied for scratch resistance tests.

**Figure 11:**
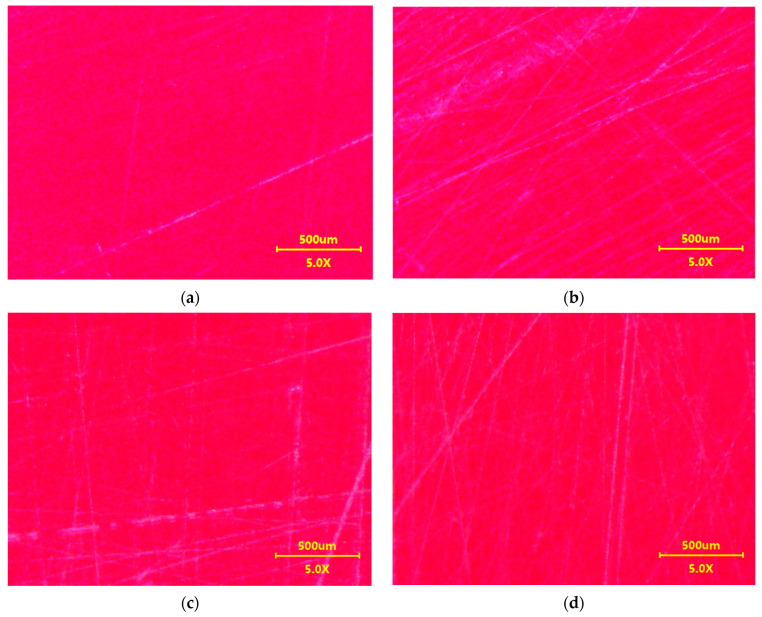
Microscopic images of the surface of R samples used for the scratch resistance test: (**a**) R#6 new, (**b**) R#6-rubbed 10 times through filter paper, (**c**) R#4–8 days washed with Turbo Active Green solution and rubbed daily through filter paper, (**d**) R#5–8 days washed with Permeate 50% solution and rubbed daily through filter paper.

It has been documented that the protective coating (clearcoat) applied to improve appearance also provides scratch resistance of automotive coatings [24,32]. Degradation of the clearcoat layer reduces abrasion and scratch resistance [22]. It was assumed that if the reclaimed water tested accelerates paint degradation, it also reduces scratch resistance. For comparison, samples taken from car doors were also washed with a 0.5% Turbo Active Green solution. The tested samples were wetted with isopropanol, and then their surface was rubbed several times with the use of filter paper. Its surface is uneven (Figure 10) and contains various minerals in addition to cellulose [21], which can cause scratches on the clearcoat surface. Rubbing the non-washed sample (Figure 11a) through the filter paper several times resulted in significant scratching of the clearcoat surface (Figure 11b). This confirms the well-known fact that automotive coatings can only be wiped with very soft materials. The damage effects of R samples treated with the use of filter paper for 8 days of washing are shown in Figure 11c (Turbo Active Green) and Figure 11d (Permeate 50%).

**Figure 12:**
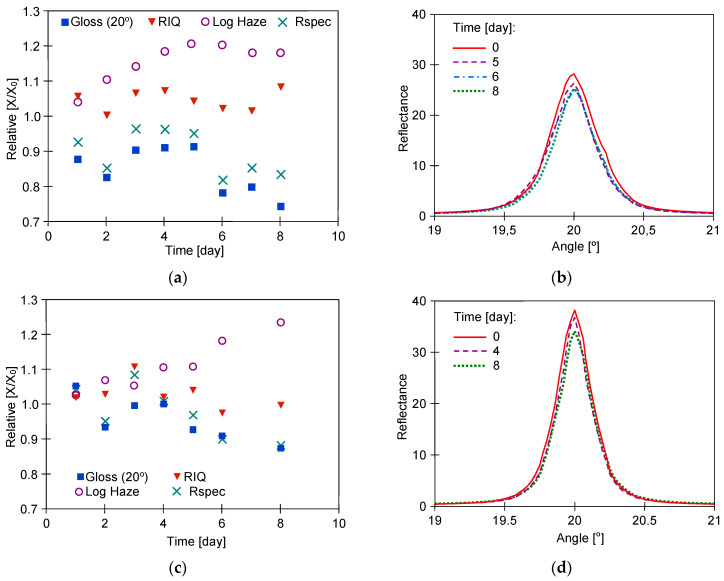
Changes of the clearcoat parameters during the scratch test with 8 days washing by 0.5% solution of Turbo Active Green-sample R#4 (**a**,**b**) and Permeate 50%-sample R#5 (**c**,**d**).

The results of microscopy observations did not show that the use of reclaimed water increased the amount of scratching (Figure 11). Furthermore, the degree of surface damage on the washed samples was similar to the scratching level noted for the non-washed sample (Figure 11b). However, measurements with the Rhopoint IQ apparatus showed that there was a decrease in the quality of the clearcoat surface on the following test day (Figure 12). As a result of the resulting scratches, the gloss value for the sample washed with Turbo Active Green solution decreased by 30 per cent and for the reclaimed water by 15 per cent. The height of the goniophotometric curves decreased with measurement time, which also indicates an increase in clearcoat turbidity (Figure 12b,d). Although both samples were taken from the same car door, the reflectance profiles for samples R#4 washed by Turbo Active Green were lower and wider (Figure 12b) than observed in Figure 12d (Permeate 50%). This was due to the fact that the surface of sample R#4 was of poorer quality, as indicated by the slightly poorer initial parameters (Table 2).

## 4. Conclusions

The alkaline solution of Active Turbo Green (pH = 8.7), used at car washes, and UF permeates (pH = 8.5–8.6) obtained from wastewater collected at the car wash, were used to test the resistance of automotive coatings to washing water. In the study conducted, car body samples were in contact with the washing water for 8 days. The results showed that the effects of the reclaimed water (UF permeates) were similar to those obtained for the washing solution used at professional car washes.

The gloss of new car coatings is generally in excess of 90 GU. After 4–5 years of car use, due to environmental degradation, it has decreased to a level of 70–80 GU. The actions of the reclaimed washing water tested only slightly altered this value and, in some cases, improved the gloss and haze parameters. Assuming that the dosing time of the solutions at the car wash does not exceed 20 min, a period of 8 days corresponds to washing the car 600 times. In reality, during service life, cars are washed much less frequently; thus, it can be assumed that washing cars at a touchless car wash does not impair the quality of the automotive coatings, even when reclaimed water is used.

## Figures and Tables

**Table 1:** Washing water parameters. Wastewater collected from the touchless car washes.

Parameter	Permeate 50%	Permeate 75%	T. Active Green ^1^	Wastewater ^2^
COD [mg/L]	535 ± 2.5	565 ± 0.5	2298 ± 13	1018 ± 10
BOD [mg/L]	237 ± 2	243 ± 2	690 ± 1	369 ± 1
total N [mg/L]	6.64 ± 0.02	8.27 ± 0.03	8.48 ± 0.02	14.4 ± 0.04
total P [mg/L]	5.26 ± 0.1	6.13 ± 0.07	0	12.5 ± 0.09
anionic [mg/L]	75.7 ± 0.7	79.6 ± 0.2	691 ± 11	135 ± 10
nonionic [mg/L]	3.46 ± 0.1	4.17 ± 0.06	30 ± 1.6	14.3 ± 1.6
pH [-]	8.5 ± 0.1	8.6 ± 0.1	8.7 ± 0.1	8.6 ± 0.1

^1^ T. Active Green—0.5% solution of Turbo Active Green Foam cleaning agent; ^2^ wastewater—used as a feed for UF process.

**Table 2:** The initial and final parameters of study samples. Average values for a sample measured 30 times. 8d—results after 8 days of washing the samples. Gloss [GU]—measured at 20°, 60° and 85°.

Sample	Gloss 20°	Gloss 60°	Gloss 85°	RIQ	Log Haze	Rspec
R#1	71.8 ± 12.2	86.3 ± 1.6	96.6 ± 2.8	57.8 ± 13.4	48.7 ± 5.8	36.5 ± 7.8
8d–T.A.G. ^1^	65.9 ± 3.9	82.5 ± 0.4	96.8 ± 0.3	54.3 ± 5.4	51.5 ± 3.8	31.3 ± 3.1
R#2	66.2 ± 2.5	82.4 ± 1.6	88.8 ± 7.3	55.9 ± 5.3	47.3 ± 7.6	32.7 ± 2.3
8d–P.50% ^2^	64.0 ± 8.3	82.6 ± 1.3	87.9 ± 1.9	51.8 ± 8.5	42.9 ± 2.9	30.8 ± 4.7
R#3	74.1 ± 0.7	87.1 ± 0.3	96.2 ± 0.4	58.9 ± 6.6	49.0 ± 2.9	38.2 ± 3.8
8d-P.75% ^3^	69.6 ± 3.3	82.1 ± 1.5	96.7 ± 0.5	60.1 ± 5.5	60.8 ± 4.0	36.2 ± 3.4
R#4	70.1 ± 1.4	83.8 ± 0.7	96.3 ± 0.4	49.1 ± 3.5	69.7 ± 7.4	30.6 ± 1.4
8d-scratch	55.7 ± 6.4	75.9 ± 2.2	95.8 ± 0.5	49.7 ± 4.8	79.5 ± 12.6	26.1 ± 3.5
R#5	74.0 ± 2.3	87.2 ± 0.9	96.2 ± 2.5	58.9 ± 3.9	49.0 ± 3.5	38.2 ± 2.6
8d–scratch	60.4 ± 2.6	79.9 ± 1.2	97.1 ± 0.5	62.9 ± 7.0	74.2 ± 7.7	33.6 ± 3.6
R#6	64.3 ± 3.6	84.0 ± 1.4	95.8 ± 0.4	55.8 ± 3.6	77.0 ± 6.1	31.7 ± 2.1
only scratch ^4^	63.1 ± 6.7	79.4 ± 2.4	96.4 ± 0.6	52.4 ± 6.4	79.4 ± 9.8	29.4 ± 3.4
W#1	78.4 ± 1.4	93.1 ± 0.9	96.2 ± 0.4	44.6 ± 4.3	48.8 ± 4.8	32.0 ± 1.9
8d–T.A.G.	81.3 ± 2.1	91.6 ± 1.0	97.5 ± 0.6	44.5 ± 6.6	46.5 ± 4.7	33.1 ± 2.8
W#2	77.8 ± 4.6	85.7 ± 1.9	93.6 ± 0.5	45.9 ± 6.2	64.9 ± 8.6	27.9 ± 1.4
8d–P.50%	81.6 ± 1.6	94.1 ± 0.4	96.8 ± 0.4	44.4 ± 4.2	49.5 ± 6.1	33.4 ± 2.6
W#3	82.3 ± 0.8	93.1 ± 0.5	96.2 ± 0.7	45.8 ± 3.8	57.4 ± 2.2	33.4 ± 1.7
8d-P.75%	82.8 ± 1.7	92.1 ± 0.4	95.8 ± 1.3	45.9 ± 5.9	56.5 ± 6.1	33.3 ± 2.7
W#4	71.7 ± 3.8	87.9 ± 1.7	96.9 ± 0.2	31.2 ± 5.8	75.2 ± 13.1	22.7 ± 3.1
CH#1	76.6 ± 16.4	86.9 ± 1.6	96.5 ± 2.5	74.4 ± 13.5	13.1 ± 4.5	51.9 ± 11.7
8d–T.A.G.	81.4 ± 1.1	91.7 ± 0.9	91.6 ± 2.5	72.3 ± 4.5	10.1 ± 1.4	52.1 ± 3.9
CH#2	83.1 ± 0.8	91.1 ± 0.7	93.2 ± 0.8	70.1 ± 7.4	11.5 ± 1.9	54.4 ± 6.6
8d–P.50%	79.6 ± 8.3	88.9 ± 1.1	88.2 ± 4.6	62.9 ± 9.8	10.1 ± 0.7	45.5 ± 4.7

^1^ T.A.G.—Turbo Active Green. ^2^ P.50%—Permeate 50%. ^3^ P.75%—Permeate 75%. ^4^ only scratch—new sample, wiped only 10 times with paper (not exposed to wash).

**Table 3:** Tortuosity parameters: Sq–root mean square height, Sa–arithmetic mean height.

Parameter	Non washed	Permeate 50%	Permeate 75%	T. Active Green
R-Sq	3.07 ± 0.33	2.81 ± 0.22	1.63 ± 0.47	2.93 ± 0.41
R-Sa	2.33 ± 0.27	2.12 ± 0.14	1.41 ± 0.32	1.99 ± 0.25
W-Sq	4.94 ± 0.15	3.51 ± 0.50	3.42 ± 0.17	3.24 ± 0.11
W-Sa	3.81 ± 0.19	2.49 ± 0.48	2.41 ± 0.12	2.08 ± 0.09
CH-Sq	8.51 ± 2.74	2.81 ± 0.22	-	3.16 ± 0.03
CH-Sa	5.27 ± 1.34	2.12 ± 0.14	-	1.74 ± 0.02

## Data Availability

The original contributions presented in the study are included in the article, further inquiries can be directed to the corresponding author.
